# Delivering mental healthcare to patients with a depressive disorder alongside a life-limiting illness

**DOI:** 10.1192/bjb.2021.124

**Published:** 2023-02

**Authors:** Daniel Hughes, Sarah Yardley, Philippa Greenfield, Martin Rolph

**Affiliations:** 1 Camden and Islington NHS Foundation Trust, UK; 2 University College London, UK; 3 Cardiff, UK

**Keywords:** Depressive disorders, anxiety disorders, antidepressants, education and training, service users

## Abstract

The concurrent assessment and treatment of mental health disorders and palliative illnesses is complex. Affective disorders are more prevalent in people who need palliative care. Identifying the most suitable place of care and multi-professional multidisciplinary teams to provide support can be challenging and bewildering for professionals and patients. Mental health clinicians may be left with a sense of therapeutic nihilism, while palliative care teams can feel limited by the mental health resources available for treating those living with significant physical and mental health needs. We discuss the fictional case of a gentleman with metastatic bowel cancer who has developed symptoms of depressive disorder and identify how taking a pragmatic patient-centred approach can offer a route through potential dilemmas when seeking to provide individualised care based on needs. We used lay person experience alongside our own experiences of novel mechanisms for cross-specialty working in order to direct psychiatric trainees’ approaches to such cases.

## Clinical scenario

You are the psychiatric trainee within a general adult psychiatry community team. A community palliative care clinical nurse specialist (CNS) phones you with concerns about Jeremy, a 50-year-old with lung metastases from bowel cancer, for which he no longer receives oncological treatment. He has no history of mental illness. Jeremy describes feeling low and not enjoying his previous passions. His sleep is poor, waking after a few hours. Energy and concentration are low, which Jeremy ascribes to his medications. Recent blood tests show no metabolic abnormalities. Jeremy has not started any new medications.

Jeremy didn't attend the initial clinic appointment. Following your multidisciplinary team (MDT) meeting discussions, you agree to assess him at home with his wife, Caroline. Jeremy explains that he is not fearful about dying (‘what choice do I have?’) but worries about the strained relationship with his adult son, who is unaware of the seriousness of his condition. Jeremy says he has recently been preoccupied with thoughts about his late father, a man who was physically violent. Jeremy mentions that he often thinks about taking an overdose to ‘speed things along’ but worries about the impact on Caroline. Jeremy appears to be experiencing a depressive episode.

## Psychiatric trainee perspective

You might feel Jeremy does not meet the threshold for secondary care mental health services and are not sure how useful your one-off home visit is. You worry that Improving Access to Psychological Therapies (IAPT) might not accept someone with significant physical health problems, or that their waiting list will be too long. Not having managed patients with palliative diagnoses much, you feel unsure about prescribing – is there any evidence that antidepressants work at end of life? You advise that Jeremy is reviewed by his general practitioner (GP) and referred back to mental health services if there are concerns about increased risk, but you think that this is unsatisfactory and of little to no benefit for a patient who is being passed between services. You plan to discuss Jeremy in your next supervision and bring his case back to the MDT.

## Palliative care CNS perspective

The CNS is frustrated. Her caseload has many patients with psychiatric problems for whom it is very difficult to get support. She does not think that the palliative care team has the right experience or skills to manage patients with suicidal thoughts and worries that one of her patients will end their own life. In this case, the palliative care consultant is reluctant to prescribe antidepressants, and the GP says that they might interact with other medications that Jeremy takes. From the CNS's perspective, multiple doctors including a psychiatrist have assessed Jeremy without offering much intervention, and it seems it is going to be left to the CNS to support him. Although she feels comfortable talking about death and dying, she feels out of her depth talking about suicide. The challenges such a case highlights are summarised in Box 1.

Box 1 Summary

- How common are affective disorders in a palliative population?
- How should the assessment and treatment of affective disorders in this population be approached?
- What are the relevant differential diagnoses?
- What are the relevant prescribing issues when treating mental illness in palliative care?
- Is there any evidence for psychological therapies at the end of life? Where can patients access these?
- How common are suicidal thoughts at the end of life? And how should we assess and alleviate these?
- What service models might improve the mental health of people with life-limiting illnesses?

## Discussion

### Affective and anxiety disorders in patients who need palliative care

There is increasing evidence of higher rates of psychiatric disorder in people receiving palliative care. Published rates of depression in advanced cancer vary from 3 to 77%, with anxiety disorders having a similar wide range in prevalence.^1,2^ Patients with advanced chronic obstructive pulmonary disease demonstrate depression in 37–71% of cases, while those with advanced heart disease report depression in 9 to 36%.^3^ The majority of those attending community cancer clinics who also have a diagnosis of depression are undertreated with regard to their mental health.^3^

Diagnosing mental illness at end of life poses significant challenges. Adjusting to death can be associated with significant anxieties.^4^ The ICD diagnostic criteria describe adjustment disorder as a ‘[state] of subjective distress and emotional disturbance … arising in the period of adaptation to a significant life change or to the consequences of a stressful life event’.^5^ It is easy to see how this may apply to patients at the end of life.

Depression in those with life-limiting illnesses has been subject to more research than other mental illnesses and has been demonstrated in 24.6% of such patients.^2^ Having depression alongside a palliative diagnosis may reduce life expectancy.^6^ A patient may describe subjectively feeling ‘low’ for multiple reasons. Differentiating between normal sadness, normal grief responses, adjustment disorder and major depressive disorder is difficult. We reiterate, however, that depression is known to be under-recognised and under-treated in palliative populations.^7^

Anxiety is a normal response to feeling under threat and an expected response to receiving ‘bad news’ or approaching end of life. One study found that 13.9% of those receiving palliative care met the criteria for an anxiety disorder^8^ without mirroring the same risk factors as the general population.^9^

A trauma-informed approach to palliative patient care highlights the abundance of potentially psychologically traumatising events which often occur preceding a patient becoming palliative, including intensive medical interventions alongside escalating illness. Prior trauma may be reactivated by the stress of approaching end of life.^10^ Receiving a palliative diagnosis may be accompanied by a strong feeling of ‘loss of control’, which can reactivate prior trauma. As such, it is important to be mindful of trauma-related symptoms.

### A wish to hasten death and suicidal thoughts: a spectrum?

Any person at the end of life may report a wish to die, or hasten death, in either the presence or absence of mental disorder. The reasons behind such wishes are multifactorial and include a direct response to physical or psychological suffering, loss of self, fear of dying, a method of ending suffering and a form of control over one's life.^11^ Discussing such a wish to hasten death does not necessarily cause distress, and patients may find it beneficial.^12^ The point at which a wish to hasten death becomes an active suicidal thought can be difficult to define, particularly in those with concurrent mental health problems. It is likely that such thoughts exist upon a spectrum. While a wish to hasten death tends to fluctuate in intensity and frequency, those with a diagnosis of depression may need tailored interventions to achieve relief, highlighting the importance of accurate diagnosis.^13^

Despite increased rates of depression in those receiving palliative care, suicide risk appears low,^14^ including for those cared for at home^15^ and in hospices.^16^ It may be that deteriorating physical health makes suicide practically difficult or that there is a fundamentally different psychological response with a lessened propensity to act. It is also possible that suicide is difficult to determine as cause of death and so is underreported.

### Diagnosing affective disorders in palliative populations

Diagnosing any affective disorder in this population is a significant challenge. It is important to first exclude any physical cause of suspected psychiatric symptoms, for example, considering hypoactive delirium in a patient who is flat and withdrawn. Taking a standardised psychiatric interview approach (asking about sleep, appetite, energy, etc.) may result in misdiagnosing an affective disorder in those experiencing predominantly physical health problems. Indeed, such somatic symptoms have been demonstrated to have a low positive predictive value for diagnosing depression in palliative care.^17^ Metabolic disturbances (such as hypercalcaemia) can cause low mood, and frequently used medications such as steroids can result in affective disturbance. A comprehensive assessment of both physical and mental health is paramount. For example, are untreated physical symptoms contributing to anxiety or poor sleep? Psychosocial considerations should also be included in any psychiatric formulation – for instance, unresolved relationship issues might emerge as death approaches.

The patient may be primarily seen by palliative care teams, who might not feel confident in diagnosing mental disorders. It can be difficult to determine what is ‘normal’ and what is ‘abnormal’. Various diagnostic screening approaches have been trialled, from single-item questionnaires to semi-structured psychiatric interviews, with similar levels of validity.^18^ Ultimately, suspecting or making a diagnosis may be limited by any perceived lack of treatment options or appropriate referral routes.

## Treatment approaches

Before starting any treatment, consider monitoring a patient's mental state over a period of time and optimising their palliative care, in order to see whether physical symptom reduction results in improvement in mood. However, such a ‘watch and wait’ approach can be challenging given time limitations.

Any patient's specific circumstances (including their primary physical diagnosis) must be considered when prescribing psychotropic medication. Pharmacological treatment must pay particular attention to the pharmacokinetics (‘what the body does to the drug’) and pharmacodynamics (‘what the drug does to the body’).^19^ Drug absorption, protein binding, metabolism and excretion are often impaired in patients receiving palliative care, and it is important to consider renal and hepatic function.

Prescribing should ascribe to the ‘start low and go slow’ orthodoxy to minimise risk, although regular review may allow for faster titration if tolerated and avoid the risk of underdosing. Drug interactions are common.^17^ Whereas an antidepressant with a long half-life such as fluoxetine can be more forgiving of a patient forgetting doses, a shorter-acting medication such as mirtazapine is easier to stop if there are serious adverse effects. It would be preferable for any treatment to be fast-acting. It is important to consider risk of prolonged QTc with certain antipsychotics (such as haloperidol) or bleeding risk with selective serotonin reuptake inhibitors (SSRIs). Such considerations are even more pertinent in this population owing to their overall frailty.

The European Palliative Care Research Collaborative recommends citalopram or mirtazapine as first-line antidepressant agents,^20^ although the Palliative Care Adult Network Guidelines highlight that there is ‘little to choose’ between SSRIs and that consideration should be given to using medications already being prescribed for other symptoms, such as tricyclic antidepressants.^21^

A 2011 systematic review concluded that ‘[antidepressants] were effective in treating depression in palliative care but it was possible that efficacy may have been overestimated due to selective reporting and publication biases’.^22^ Studies looking at antidepressant use in other (non-palliative) physical health conditions may give more reason to be optimistic.^23^ Research involving other psychotropic medications, including benzodiazepines, antipsychotics and psychostimulants, provides even less evidence for these than for traditional antidepressants.

Psychological approaches to affective disorders in people receiving palliative care have similar research difficulties, although specific therapies such as Dignity Therapy^24^ and group therapy utilised in metastatic breast cancer^25^ have demonstrated some efficacy. More generally, there is some evidence for cognitive–behavioural therapy (CBT) to treat mood disorders in those receiving end-of-life care.^26,27^ There is an argument for integration of psychological therapies within palliative care teams, with evidence that CBT delivered by palliative care nurses can reduce anxiety levels.^28^

Managing suicide risk includes assessing potential access to large amounts of prescribed medication. We suggest involvement of mental health crisis teams alongside community palliative care teams as an effective way to formulate crisis plans. Less restrictive options should be fully explored, and practically a palliative diagnosis may fundamentally change the approach to suicide risk. Admitting a patient to a psychiatric hospital for short-term risk containment seems rarely appropriate as someone approaches the end of life.

All psychiatric treatment should be offered in conjunction with ongoing comprehensive palliative care, ideally via joint reviews. Such working is an excellent opportunity to gain from sharing clinical expertise and allows opportunities for patient-centred treatment.

### Reflection: challenges and opportunities

People who need palliative care may not fit into traditional psychiatric service models, particularly when these are designed with inflexible diagnostic criteria, envisaging the patient as a largely passive recipient of treatment, rather than integrating the benefits of co-production. Although IAPT does accept patients for treatment with physical health needs, the complexities of people under palliative care services and the importance of prompt treatment may mean that IAPT is not always best placed to deliver effective treatment. Conversely, these patients may not meet the threshold for secondary care mental health services. Their involvement with mental health services may be limited to referrals to liaison services while receiving in-patient medical care, leading to an opportunistic approach to assessment and management. Some palliative care services have access to psychologists or psychiatrists, although this is far from the norm. It may be left to the resourceful palliative care clinician to navigate their own local mental health services, to identify a ‘friendly psychiatry team’ from the range of primary care or assessment services, liaison services, community mental health services or psychological services.

Patients themselves may not want to see additional teams alongside palliative care, oncology, district nurses and general practice. It may therefore fall upon those less experienced in dealing with mental illness to offer informal psychological support. Palliative care clinicians are experienced at ‘sitting with’ patient distress, an approach that lends itself well to managing psychiatric disorders. In return, mental health services need to act in a more flexible manner than that to which they are perhaps accustomed, for example, accepting people receiving palliative care for assessment who would otherwise not reach their service threshold.

### A model of integrated care: embedding a psychiatric trainee within a palliative care community team

It may be that mental health services can also offer a role in supporting palliative care clinicians to feel more confident and effective in their management of mental disorder, through liaison and complex case discussion.

One approach is for a psychiatric trainee to liaise with local palliative care teams to arrange clinical work as part of special interest sessions. In response to a local audit identifying substantial unmet mental health needs within a community palliative care team,^29^ the authors embedded a psychiatric trainee within this team. We proposed this would improve psychiatric assessment and mental health treatment signposting, facilitate training of palliative care staff in assessing mental health, and improve the physical health knowledge of the psychiatric trainee. The trainee joined MDT meetings, advised team members and attended joint reviews of patients with potentially unmet psychiatric needs.

This intervention had a positive impact upon the number of cases that had to be brought to MDT discussions each month with psychiatric problems. Prior to psychiatric trainee involvement, a mean of 2.3 new cases per month were considered to involve unmet mental health needs, and a mean of 5.6 ‘complex’ cases involving unmet mental health needs were discussed each month. During the intervention, these means reduced to 1.3 (new patients) and 3.6 (complex patients).

The psychiatry trainee found this an invaluable experience to develop understanding of the complexities of treating mental health problems alongside palliative diagnoses. Furthermore, palliative care clinicians may benefit significantly from exposure to mental health services and approaches being incorporated into their work.

### Family experience

Caroline has worried about her husband for months. He seems withdrawn and not interested in doing things that he used to enjoy. Caroline tried to persuade Jeremy to seek help, but she felt that he was just passed between different services without anyone actually offering a diagnosis or any meaningful support. Caroline was relieved when Jeremy's CNS arranged a review with a psychiatrist. She was pleased that they were thinking about his mental health as well as his cancer.

### Patient experience

Jeremy had never experienced mental health problems before and worried about being labelled ‘mad’. He was dying, and speaking to a psychiatrist would not change that, so why bother? He had heard that antidepressants took a long time to work and might have side-effects.

Jeremy has had thoughts of ending his life, taking all the Oramorph he had been prescribed. This alarmed him – he did not want to do this to Caroline, but felt that he was starting to run out of options. Jeremy was worried that a psychiatrist might ‘lock him up’ if he discussed these thoughts, and he did not want to end his life incarcerated. Caroline convinced him to speak with a psychiatrist from the mental health team.

Although he worried about it beforehand, being able to talk through his experiences and be listened to in a non-judgemental way was helpful. Being told he was ‘depressed’ was not a surprise, but Jeremy was disappointed that there did not seem to be many services available to support him. He felt encouraged that his palliative care team were thinking about his mental health as well as his physical health. Jeremy remained unsure whether medications would be beneficial in his case, but he was later started on mirtazapine, which helped with his sleep and did lift his mood. Through his local hospice, Jeremy has been able to access some talking therapy. He was surprised when he noticed that he had become less preoccupied with his deteriorating physical health and was able to focus more on spending time doing what he enjoyed. Following talking about his difficult relationship with his son, Jeremy realised that he had been shutting him out to protect him from the seriousness of his cancer. He planned an open and honest conversation with him.

## Conclusion

Mental illness is a substantial source of morbidity for patients receiving end-of-life care and is often underdiagnosed and undertreated. Understanding how to best deliver palliative care to this patient group is of paramount importance. Services are typically delineated between ‘mental’ and ‘physical’ health, an approach that is unhelpful in delivering integrated patient-focused care^30^ and emphasises an unrealistic expectation of how people with physical comorbidities present with mental health problems and *vice versa* (i.e. whether severe mental illness precedes significant physical illness). Access to psychological support for those with very limited life expectancy remains poor.^31^ Had Jeremy been of a BAME background or spoken English as a second language, his access to mental health support may have been complicated even further (alongside difficulties in accessing palliative care).^32^ Delivery of mental healthcare to those with all physical health diagnoses, but particularly those with short prognoses, urgently needs to improve.

It can be difficult for palliative care teams to know where to get support for people living with physical and mental health needs, and there is evidence that these patients occupy a significant amount of MDT discussion time.^28^ Treating those with mental health diagnoses, particularly those with suicidal thoughts, can be distressing for all staff, and it remains important that they have appropriate support and supervision when working with this patient group. Assessing response to any intervention is difficult given time limitations and the heterogenicity of this population. It is essential that mental health services offer flexibility in approaching the care of those with both palliative and psychiatric diagnoses; realistically supporting palliative care teams to offer interventions may be the most pragmatic approach.

## About the authors

**Daniel Hughes** is an ST6 in General Adult Psychiatry at Camden and Islington NHS Foundation Trust, London, UK; **Sarah Yardley** is a Consultant in Palliative Medicine and Honorary Clinical Senior Lecturer at University College London, UK; **Philippa Greenfield** is a Consultant Psychiatrist at Camden and Islington NHS Foundation Trust, UK; **Martin Rolph** is a lay person in Cardiff, UK
